# Retraction Note: Asiaticoside, a trisaccaride triterpene induces biochemical and molecular variations in brain of mice with parkinsonism

**DOI:** 10.1186/s40035-024-00424-x

**Published:** 2024-06-05

**Authors:** Uvarajan Sampath, Vanisree Arambakkam Janardhanam

**Affiliations:** 1 Department of Biochemistry, Indo-American College, Cheyyar, Tamilnadu India; 2 Department of Biochemistry, University of Madras, Guindy Campus, Chennai, Tamilnadu India

**Retraction Note** **:** **Transl Neurodegener 2, 23 (2013)**

**https://doi.org/10.1186/2047-9158-2-23**

The Editor-in-Chief has retracted this article because concerns have been raised regarding some of the images presented in the Figures. Specifically:

- Fig. 1 all panels appear to share some highly similar features;
- Fig. 2 all panels appear to have areas of high similarity;
- Fig. 3 panels A, C and D appear to share some highly similar features;
- Fig. 4A and D appear to share some highly similar features;
- Fig. 10A and D appear to overlap;
- Fig. 10B2, 11C and D appear to overlap.

The Editor-in-Chief therefore no longer has confidence in the presented data.

Uvarajan Sampath does not agree to this retraction. Vanisree Arambakkam Janardhanam has not responded to correspondence from the publisher about this retraction notice.

